# The retromer CSC subcomplex is recruited by MoYpt7 and sequentially sorted by MoVps17 for effective conidiation and pathogenicity of the rice blast fungus

**DOI:** 10.1111/mpp.13029

**Published:** 2020-12-21

**Authors:** Congxian Wu, Yahong Lin, Huawei Zheng, Yakubu Saddeeq Abubakar, Minghui Peng, Jingjing Li, Zhi Yu, Zonghua Wang, Naweed I. Naqvi, Guangpu Li, Jie Zhou, Wenhui Zheng

**Affiliations:** ^1^ Key Laboratory of Pathogenic Fungi and Mycotoxins of Fujian Province College of Life Sciences Fujian Agriculture and Forestry University Fuzhou China; ^2^ Institute of Oceanography Minjiang University Fuzhou China; ^3^ Department of Biochemistry Faculty of Life Sciences Ahmadu Bello University Zaria Nigeria; ^4^ State Key Laboratory of Ecological Pest Control for Fujian and Taiwan Crops College of Plant Protection Fujian Agriculture and Forestry University Fuzhou China; ^5^ Temasek Life Sciences Laboratory, and the Department of Biological Sciences National University of Singapore Singapore Singapore; ^6^ Department of Biochemistry and Molecular Biology University of Oklahoma Health Sciences Center Oklahoma City Oklahoma USA

**Keywords:** *Magnaporthe oryzae*, pathogenicity, recruitment, retromer, sorting

## Abstract

In eukaryotic cells, Rab GTPases and the retromer complex are important regulators of intracellular protein transport. However, the mechanistic relationship between Rab GTPases and the retromer complex in relation to filamentous fungal development and pathogenesis is unknown. In this study, we used *Magnaporthe oryzae*, an important pathogen of rice and other cereals, as a model filamentous fungus to dissect this knowledge gap. Our data demonstrate that the core retromer subunit MoVps35 interacts with the Rab GTPase MoYpt7 and they colocalize to the endosome. Without MoYpt7, MoVps35 is mislocalized in the cytoplasm, indicating that MoYpt7 plays an important role in the recruitment of MoVps35. We further demonstrate that the expression of an inactive MoYpt7‐DN (GDP‐bound form) mutant in *M. oryzae* mimicks the phenotype defects of retromer cargo‐sorting complex (CSC) null mutants and blocks the proper localization of MoVps35. In addition, our data establish that MoVps17, a member of the sorting nexin family, is situated at the endosome independent of retromer CSC but regulates the sorting function of MoVps35 after its recruitment to the endosomal membrane by MoYpt7. Taken together, these results provide insight into the precise mechanism of retromer CSC recruitment to the endosome by MoYpt7 and subsequent sorting by MoVps17 for efficient conidiation and pathogenicity of *M. oryzae*.

## Graphical abstract

The retromer CSC subcomplex is recruited by MoYpt7 and sequentially sorted by MoVps17‐MoVps5 subcomplex for autophagy and endosome dynamics, which mediate the effective conidiation and pathogenicity of *Magnaporthe oryzae*.

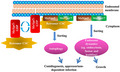

## 1 INTRODUCTION

Vesicle transport exists in all eukaryotic cells to mediate material and signal communication, and it involves defined and dynamic space–time coupled processes regulated by many different proteins (Faini et al., 2013; Jahn & Scheller, 2006; Seaman, 2008). Rab GTPases are among the essential components and regulators of vesicle transport that mediate various steps of membrane trafficking (Hutagalung & Novick, 2011; Li & Marlin, 2015; Pfeffer, 2013; Wandinger‐Ness & Zerial, 2014). They are evolutionarily conserved in many organisms ranging from yeast to humans, and function as molecular switches by alternating between inactive (guanosine diphosphate [GDP]‐bound) and active (guanosine triphosphate [GTP]‐bound) states to promote vesicle formation, scission, movement, and fusion at the target membrane (Hutagalung & Novick, 2011; Li & Marlin, 2015; Pfeffer, 2013; Wandinger‐Ness & Zerial, 2014). In particular, Rab7 is a conserved protein that plays an important role in the late endocytic pathway and in lysosome biogenesis in mammalian cells (Chavrier et al., 1990; Modica et al., 2017). In budding yeast (*Saccharomyces cerevisiae*) or fission yeast (*Schizosaccharomyces pombe*), the homolog of Rab7, Ypt7, is localized primarily to the vacuolar membrane and mediates docking and fusion of late endosomes with vacuoles; it is also necessary for vacuole–vacuole fusion (Balderhaar et al., 2010; Kashiwazaki et al., 2009). In *Arabidopsis*, RABG3f, a plant Rab7 homolog, localizes to prevacuolar compartments and the tonoplast, and it is involved in vacuolar trafficking, vacuole biogenesis, and plant growth (Cui et al., 2014; Ebine et al., 2014). Although the localization and functions of Rab7 have been investigated in various organisms, the molecular details of Ypt7‐mediated protein trafficking pathways in the endosomal system are still not well understood.

Retromer is a multisubunit protein transport complex that mediates transportation of various transmembrane proteins/receptors, including immune protein receptors, mannose‐6‐phosphate receptors, Shiga toxin, and Wnt receptors from early endosomes to the trans‐Golgi network (TGN) (Bonifacino & Rojas, 2006; Brown et al., 2001; Eaton, 2008; Seaman, 2005; Verges, 2007). Dysfunction of retromer abrogates various physiological processes such as secretion, storage, transport, and immune responses (Abubakar et al., 2017). Retromer is composed of two subcomplexes: a sorting nexin (SNX) dimer and a cargo‐sorting complex (CSC). The CSC consists of three vacuolar protein‐sorting (Vps) proteins, namely Vps35, Vps26, and Vps29. The C‐terminus of Vps35 binds to the phosphodiesterase‐folded region of Vps29 and its N‐terminus binds to the C‐terminal domain of Vps26, making it the central protein of the retromer complex. The SNX dimer consists of Vps5 and Vps17 proteins, which both contain PX (phox homology) and BAR (Bin‐Amphyphysin‐Rvs) domains. The PX domain binds to phosphatidylinositol on the endosomal membrane surface for effective anchorage of the retromer, whereas the BAR domain induces membrane curvature for vesicle formation (Seaman, 2005).

Studies in mammals and *S. cerevisiae* have shown that Rab7/Ypt7 coordinates retromer‐mediated cargo transport during vesicle–vacuole fusion (Balderhaar et al., 2010; Rojas et al., 2008). In mammals, GTP‐binding Rab7 protein recruits the retromer CSC to the endosomal membrane (Rojas et al., 2008). Silencing the *RAB7* gene prevents the association of the retromer CSC with the endosomal membrane, which in turn results in mis‐sorting of acid hydrolase D and inhibition of retromer‐mediated retrograde transport of cation‐independent mannose‐6‐phosphate receptor (CI‐MPR) (Rojas et al., 2008). Further study revealed that the retromer complex and Rab7 colocalize on endosomes and that GTP‐Rab7 protein interacts directly with the retromer CSC (Rojas et al., 2008). In addition, Seaman et al. showed that TBC1D5 acts as a GTPase activating protein of Rab7, which interacts with the retromer complex and negatively regulates the recruitment of retromer CSC to the endosomal membrane (Seaman et al., 2009). In yeast, the retromer CSC acts as a downstream effector of Ypt7 and overexpression of *YPT7* causes the degradation of CSC in the vacuoles, indicating that Ypt7 is essential for proper localization of the CSC (Balderhaar et al., 2010).

*Magnaporthe oryzae* is a heterozygous filamentous fungus that causes the devastating rice blast disease, which hampers rice production worldwide and threatens world food security. The organism has emerged as an important model organism for studying the development and mechanism of pathogenesis in filamentous fungi (Ebbole, 2007; Valent & Chumley, 1991). *M. oryzae* infection begins when the fungal spores land on the host surface and begin to germinate and differentiate to produce appressoria, which are required for physical penetration of the host cuticles (Mentlak et al., 2012; Veneault‐Fourrey et al., 2006; Wilson & Talbot, 2009). In our previous studies, we demonstrated that Δ*Moypt7* and Δ*Movps35* deletion mutants exhibit similar phenotypes, including defective conidiogenesis and pathogenicity (Liu et al., 2015; Zheng et al., 2015b). MoYpt7 is localized on the late endosome/vacuolar membrane and is important for the fungal growth, conidiation, vacuolization, autophagy, stress responses, and pathogenicity (Liu et al., 2015). The Δ*Movps35* mutant has delayed lipid and glycogen degradation, and is significantly reduced in conidiation and virulence on rice and barley. Further study showed that appressorial turgor pressure in the Δ*Movps35* mutant is significantly lower than that of the wild type, and autophagy is blocked during conidial germination (Zheng et al., 2015b). Both MoYpt7 and MoVps35 are important for autophagy and may work in the same trafficking pathway to regulate vegetative growth, conidiation, and pathogenesis of *M. oryzae*.

However, our understanding of the relationship between Rab GTPases and the retromer complex in filamentous fungi is completely unknown. In this study, we dissect the molecular interactions and functional mechanisms of these two intracellular trafficking machineries in *M. oryzae*. Using affinity purification/mass spectrometry assay, coimmunoprecipitation (Co‐IP), and colocalization studies, we show that MoYpt7 interacts with MoVps35, the core subunit of the retromer complex. Loss of MoYpt7 results in mislocalization of MoVps35 to the cytoplasm, which reduces *M. oryzae* conidiation and pathogenesis. Furthermore, MoVps35 is recruited to the late endosomes by MoYpt7 in a nucleotide‐dependent manner. Finally, we show that the retromer‐sorting nexin protein MoVps17 replaces MoYpt7 for sequential sorting of retromer CSC in pathogenicity of the rice blast fungus. Our data address the key question of how a plant pathogen balances vesicular recruitment and subsequent sorting events to govern its development and pathogenesis.

## 2 RESULTS

### 2.1 Punctate MoVps35 associates with MoYpt7 on late endosomal and vacuolar membranes

**FIGURE 1:**
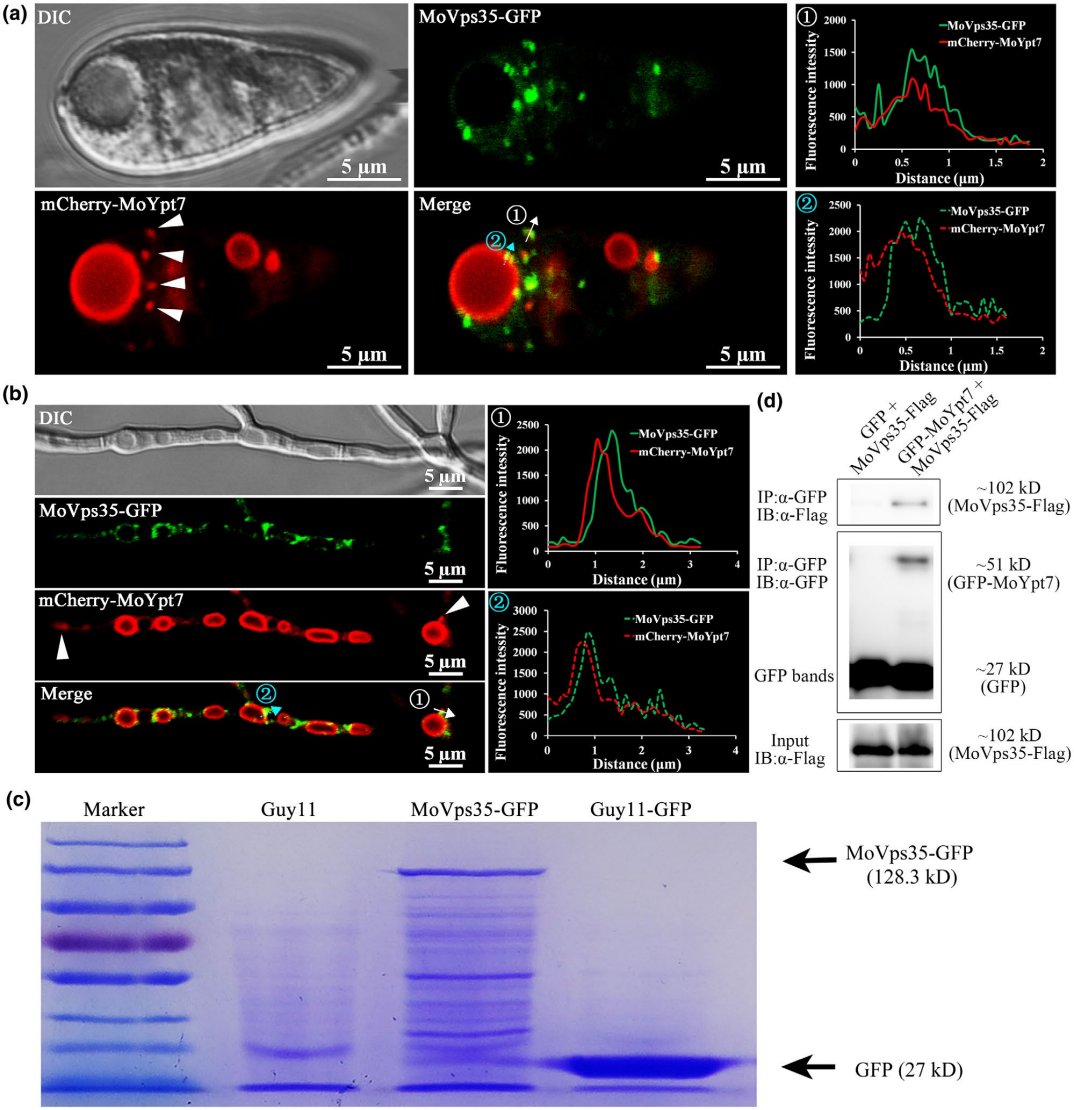
Interaction and colocalization of MoYpt7 with MoVps35. (a, b) mCherry‐MoYpt7 colocalizes with MoVps35‐GFP in the conidia and mycelia of Guy11. The mCherry‐MoYpt7 appears on vacuolar membrane and dotted endosome (white arrowheads) in both conidia and mycelia. The line scan graphs confirm the colocalization of MoVps35‐GFP and mCherry‐MoYpt7 in the conidia and mycelia of *Magnapothe oryzae*. ① indicates that both MoVps35‐GFP and mCherry‐MoYpt7 colocalized on the punctate endosome.② indicates that MoVps35‐GFP associated with the mCherry‐MoYpt7‐marked vacuolar membrane. Bar = 5 μm. (c) SDS‐polyacrylamide gel electrophoresis (SDS‐PAGE) image of protein bands of eluted samples of GFP‐Trap beads. Guy11 represents the wild‐type strain, MoVps35‐GFP represents the Δ*Movps35* mutant expressing the MoVps35‐GFP construct, Guy11‐GFP represents a wild‐type strain expressing free green fluorescent protein (GFP). (d) Coimmunoprecipitation analysis showing the interaction of MoYpt7 with MoVps35. GFP control or GFP‐MoYpt7 were coexpressed with *MoVps35‐FLAG* vector in Guy11 strain. Total proteins were extracted from the successful transformants and GFP‐Trap beads were used to pull down GFP‐MoYpt7 protein; its interacting proteins were then separated by SDS‐PAGE. The GFP‐MoYpt7 and MoVps35‐FLAG proteins were identified by α‐GFP and α‐FLAG antibodies, respectively

MoYpt7 is mainly localized on late endosomal and vacuolar membranes, while MoVps35, a core subunit of the retromer complex, appears as fast‐moving punctate structures close to FM4‐64‐marked vacuolar membranes in *M. oryzae* (Liu et al., 2015; Zheng et al., 2015b). We therefore investigated the possible colocalization of the two proteins. mCherry‐MoYpt7 and MoVps35‐GFP were coexpressed in the Guy11 strain via transformation of the respective plasmid constructs, and their intracellular localization was examined by laser scanning confocal microscopy. We found that MoYpt7 appeared on ring‐shaped vacuoles and dotted structures in both mycelial and conidial cells, while MoVps35 exhibited its usual punctate appearance inside the cells (Figure 1a,b). Strikingly, the majority of MoVps35 punctate structures were aligned at the surface of the MoYpt7 vacuoles and dotted endosomes, and this was further verified by line scan assays (Figure 1a,b). These data clearly demonstrate that MoVps35 closely associates with MoYpt7 on the late endosomal and vacuolar membranes.

### 2.2 MoVps35 interacts with MoYpt7 in *M. oryzae*

The partial colocalization of MoVps35 and MoYpt7 prompted us to check whether there is direct interaction between the two proteins. We first expressed MoVps35‐GFP in the Δ*Movps35* strain and then used GFP‐Trap beads to extract MoVps35‐GFP and its associated proteins in pull‐down assays. Considering the fact that MoYpt7 and retromer complex have both been found to be involved in autophagy, which is essential for *M. oryzae* conidiogenesis and pathogenicity (Liu et al., 2015; Zheng et al., 2015b), we analysed the MoVps35 interactomes obtained from nutrient‐sufficient and nitrogen‐deficient conditions for uninduced and induced autophagy, respectively. Two negative control strains, Guy11 (wild type) and Guy11‐GFP (wild type expressing green fluorescent protein, GFP), were introduced for parallel analysis. Proteins bound to the GFP‐Trap beads were eluted and fractionated by 10% sodium dodecyl sulphate (SDS) polyacrylamide gel electrophoresis (PAGE) and stained with Coomassie brilliant blue R‐250. As shown in Figure 1c, many protein bands were observed in the MoVps35‐GFP strains as opposed to only a few in the two negative controls (Guy11 and Guy11‐GFP). Digestion with trypsin and subsequent liquid chromatography‐tandem mass spectrometry (LC‐MS/MS) analyses revealed that the retromer CSC subcomplex, MoVps35‐MoVps26‐MoVps29, was repeatedly captured as one of the interacting proteins in all the replicates of MoVps35‐GFP pull‐down (Table 1), and this was considered as high‐confidence positive control. Remarkably, MoYpt7 was also captured repeatedly in the MoVps35‐GFP pull‐down assays, suggesting a high possibility of interaction between MoYpt7 and MoVps35 (Table 1).

**TABLE 1:** Pull‐down and mass spectrometry analyses of MoVps35‐interacting proteins

Gene	Description	Replication 1 (nutrient culture)		Replication 2 (nutrient culture)		Replication 3 (nitrogen starvation)	
		Coverage (%)	Unique peptides	Coverage (%)	Unique peptides	Coverage (%)	Unique peptides
MGG_05089 (MoVps35)	Vacuolar protein sorting‐associated protein 35	26.50	26	65.37	56	52.78	32
MGG_04830 (MoVps26)	Vacuolar protein sorting‐associated protein 26	20.82	8	66.56	23	50.47	14
MGG_02524 (MoVps29)	Vacuolar protein sorting‐associated protein 29	32.67	7	54.60	10	41.58	6
MGG_08144 (MoYpt7)	Ras‐like protein Rab7	23.41	4	43.41	7	15.61	2

Next, we tested for in vivo interaction between MoYpt7 and MoVps35 by Co‐IP. To this end, we generated a Guy11 transformant coexpressing GFP‐MoYpt7 and MoVps35‐FLAG and subjected it to immunoprecipitation (using GFP‐Trap_A beads) and subsequent immunoblot analysis (using an anti‐FLAG antibody). A 51 kDa band corresponding to GFP‐MoYpt7 and a 102 kDa band corresponding to MoVps35‐FLAG were identified, suggesting an in vivo interaction between the retromer subunit, MoVps35, and MoYpt7 (Figure 1d).

### 2.3 MoYpt7 recruits MoVps35 to the vacuolar membrane in *M. oryzae*

**FIGURE 2:**
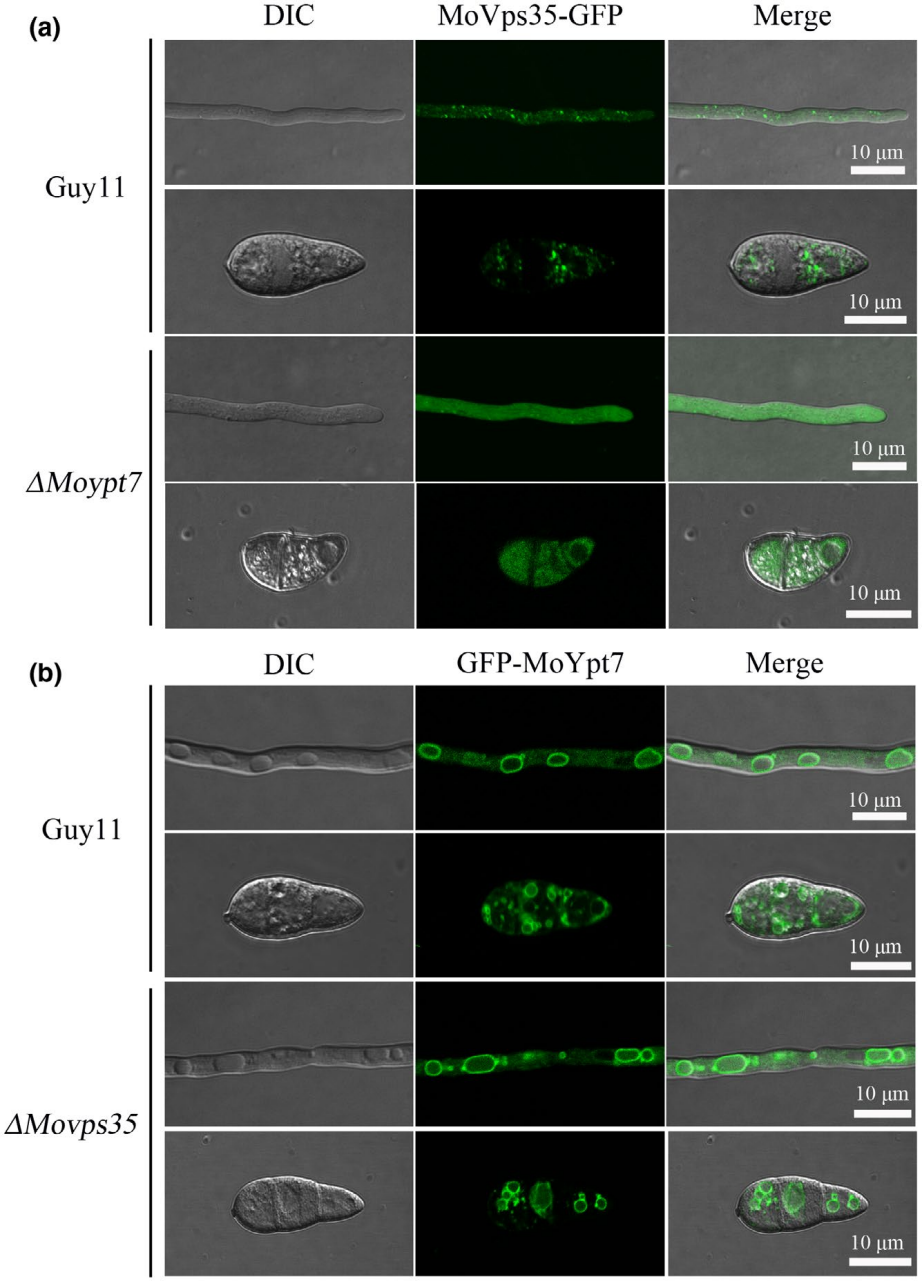
MoVps35‐GFP mislocalizes to the cytosol in the mycelia and conidia of Δ*Moypt7* mutant. (a) Localization of MoVps35‐GFP in the hyphae and conidia of the wild type Guy11 and Δ*Moypt7* mutant. Bar = 10 μm. MoVps35‐GFP expressed in Guy11 showed multiple fluorescent dots in the hyphae and conidia while MoVps35‐GFP appeared diffuse in its distribution in Δ*Moypt7*. (b) Localization of GFP‐MoYpt7 in the hyphae and conidia of wild type and Δ*Movps35* mutant. Bar = 10 μm. Deletion of *MoVps35* does not impair GFP‐MoYpt7 localization

To understand the function of MoVps35–MoYpt7 interaction, we first expressed MoVps35‐GFP in the Δ*Moypt7* mutant and determined the localization of MoVps35‐GFP. The results indicated that, in the absence of MoYpt7, MoVps35‐GFP lost its usual punctate appearance and became dispersed throughout the cytoplasm in both mycelia and conidia (Figure 2a). In contrast, when GFP‐MoYpt7 was expressed in the Δ*Movps35* mutant, the localization of GFP‐MoYpt7 remained unchanged in both mycelial and conidial cells (Figure 2b). These results suggest that MoYpt7 plays a regulatory role in the recruitment of cytoplasmic MoVps35 to the vacuolar membrane.

### 2.4 Ectopic expression of dominant negative *MoYpt7* alleles results in mislocalization of MoVps35 and subsequent defects in the fungal conidiation and pathogenicity

**FIGURE 3:**
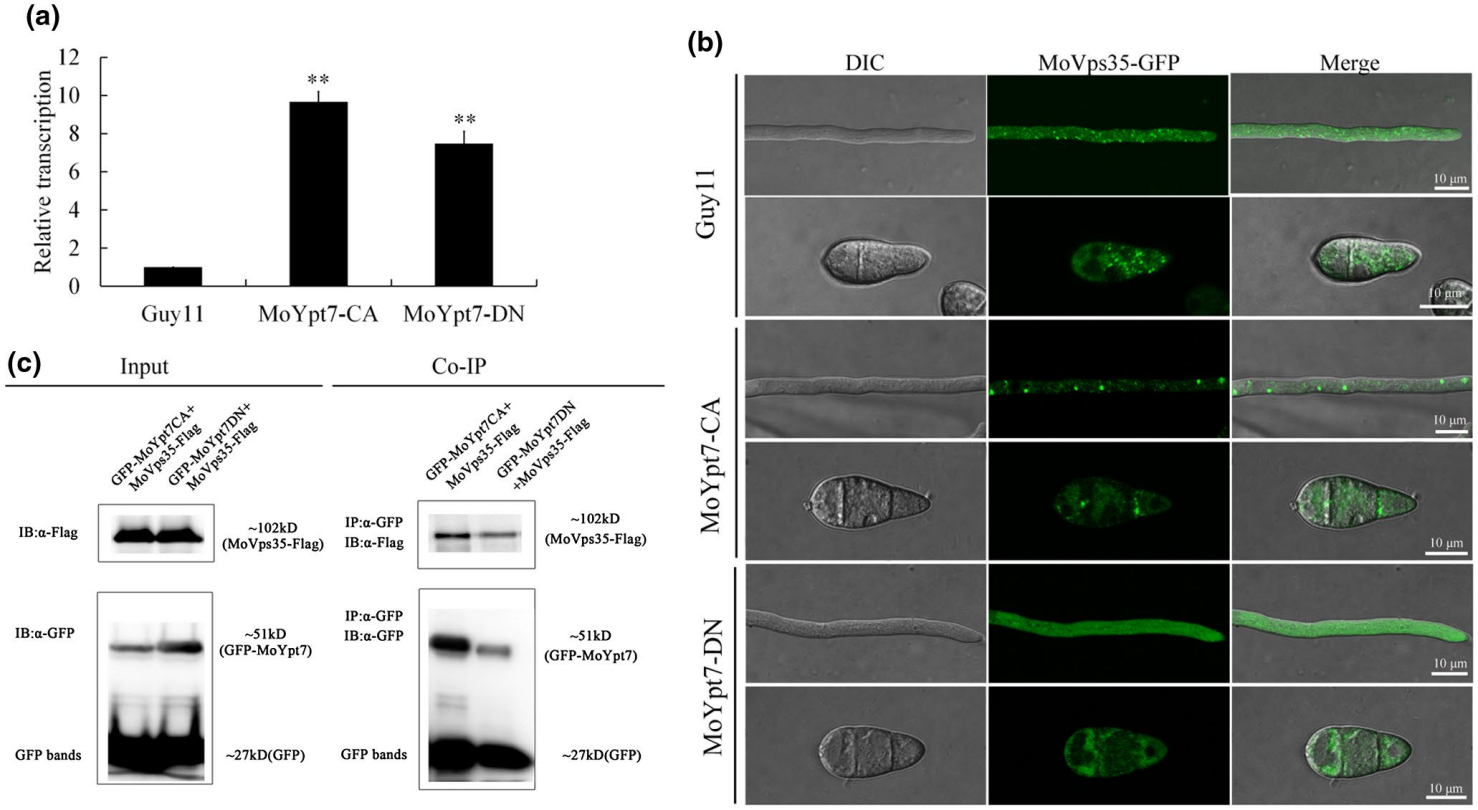
Expression level of *MoYpt7* and its activity in the normal localization of MoVps35. (a) The expression levels in the*MoYpt7‐* *CA* (constitutively active) and *MoYpt7‐* *DN* (dominant negative) strains. Standard deviations were calculated from three biological replicates. Significance was measured using an unpaired *t* test (**p < .01). (b) Localization of MoVps35‐GFP in *MoYpt7‐* *CA* and *MoYpt7‐* *DN* strains. Bar = 10 µm. Overexpressing *MoYpt7‐* *DN* in the wild type Guy11 caused MoVps35‐GFP to be unable to be targeted to the endosomes. (c) Coimmunoprecipitation analysis showed that MoYpt7‐CA and MoYpt7‐DN both interact with MoVps35. The GFP‐MoYpt7‐CA, GFP‐MoYpt7‐DN, and MoVps35‐FLAG proteins were identified by α‐GFP and α‐FLAG antibodies, respectively

Rab/Ypt family proteins change their conformations after binding a guanine nucleotide (Wandinger‐Ness & Zerial, 2014). The conformational change is regulated by a GDP/GTP exchange reaction as well as the GTPase activity (Wandinger‐Ness & Zerial, 2014). In *M. oryzae*, N125I and Q67L mutations of MoYpt7 protein favour GDP‐bound and GTP‐bound forms, respectively; these two states are important for fungal development and pathogenicity (Huang et al., 2018; Liu et al., 2015). To further investigate the regulatory role of MoYpt7 in MoVps35 localization and function, we constructed a dominant negative (DN) form of MoYpt7 (N125I mutation) and a constitutively active (CA) form of MoYpt7 (Q67L mutation). The *MoYpt7‐* *DN* and *MoYpt7‐* *CA* alleles were cotransformed with the *MoVps35‐GFP* construct into the wild‐type strain. Subsequent quantitative reverse transcription PCR (RT‐qPCR) analysis indicated that there was a more than 7‐fold increase in *MoYpt7* mRNA levels in the vegetative hyphae of *MoYpt7‐* *DN* and *MoYpt7‐* *CA* mutants as compared to the wild‐type strain in each case (Figure 3a), suggesting that the transformants expressed the expected dominant alleles of *MoYpt7*.

Next, we investigated the effects of *MoYpt7‐* *CA* and *MoYpt7‐* *DN* expression on the localization of MoVps35‐GFP in the mycelia and conidia of *M. oryzae*. In the *MoYpt7‐* *CA* strain, MoVps35‐GFP showed punctate distribution in the mycelia and conidia (Figure 3b), similar to its localization in the wild type. However, in the *MoYpt7‐* *DN* strain, MoVps35‐GFP was mislocalized and showed a dispersed fluorescence signal in the cytoplasm (Figure 3b), suggesting that MoYpt7 activity is necessary for normal localization of MoVps35. Considering the fact that Rab proteins cycle between their GDP‐bound inactive and GTP‐bound active forms between the cytoplasm and membranes (Hutagalung & Novick, 2011), we reasoned that the dispersed nature of MoVps35‐GFP into the cytoplasm in the strain expressing MoYpt7‐DN could be due to failure of MoYpt7‐DN to adhere to the late endosomal and vacuolar membranes. To test this hypothesis, *GFP*‐tagged *MoYpt7‐ DN* and *MoYpt7‐ CA* constructs were generated and transformed into the protoplasts of the wild‐type strain, and transformants were subjected to RT‐qPCR to check the expression levels of the constructs (Figures S1a and S2a). As expected, the inactive GDP‐bound form of MoYpt7 mostly localized to the cytoplasm while its active GTP‐bound form localized to vacuolar membranes in both mycelia and conidia (Figures S1b and S2b). Therefore these results indicate that MoVps35 is recruited to the late endosomes by MoYpt7 in a nucleotide‐dependent manner.

The mislocalization of MoVps35 in the *MoYpt7‐*DN mutant prompted us to investigate and compare the phenotypic features of the *MoYpt7‐*DN mutant with those of the Δ*Movps35* mutant. To this end, we examined the growth, conidiation, and pathogenicity of the *MoYpt7‐*DN mutant on barley and rice leaves. Like the Δ*Movps35* mutant (Zheng et al., 2015b), the *MoYpt7‐* *DN* mutant grew slightly slower than the wild type on various culture media, with significantly reduced conidiation and pathogenicity on plant leaves (Figure S1c–i). As compared to the wild type, the *MoYpt7‐* *CA* mutant did not show obvious reduction in growth, conidiation, and pathogenicity (Figure S2c–i). These data demonstrate that the *MoYpt7‐*DN mutant is impaired in MoVps35 recruitment and consequently mimicked the Δ*Movps35* mutant phenotype defects, including decreased conidiation and pathogenicity to the host. We further tested whether MoVps35 could act as an MoYpt7 effector for conidiogenesis and pathogenicity. If this were true, MoVps35 should physically interact with the active (GTP‐bound) MoYpt7 only and should act downstream of MoYpt7. We therefore used a Co‐IP assay to test whether the CA and the DN forms of MoYpt7 interact with MoVps35. The results clearly indicated positive interaction of MoVps35 with both the CA and the DN forms of MoYpt7 (Figure 3c), suggesting that MoVps35 is not an effector of MoYpt7.

### 2.5 Overexpression of *MoVps35* in Δ*Moypt7* fails to restore the phenotypic defects of the Δ*Moypt7* mutant

**FIGURE 4:**
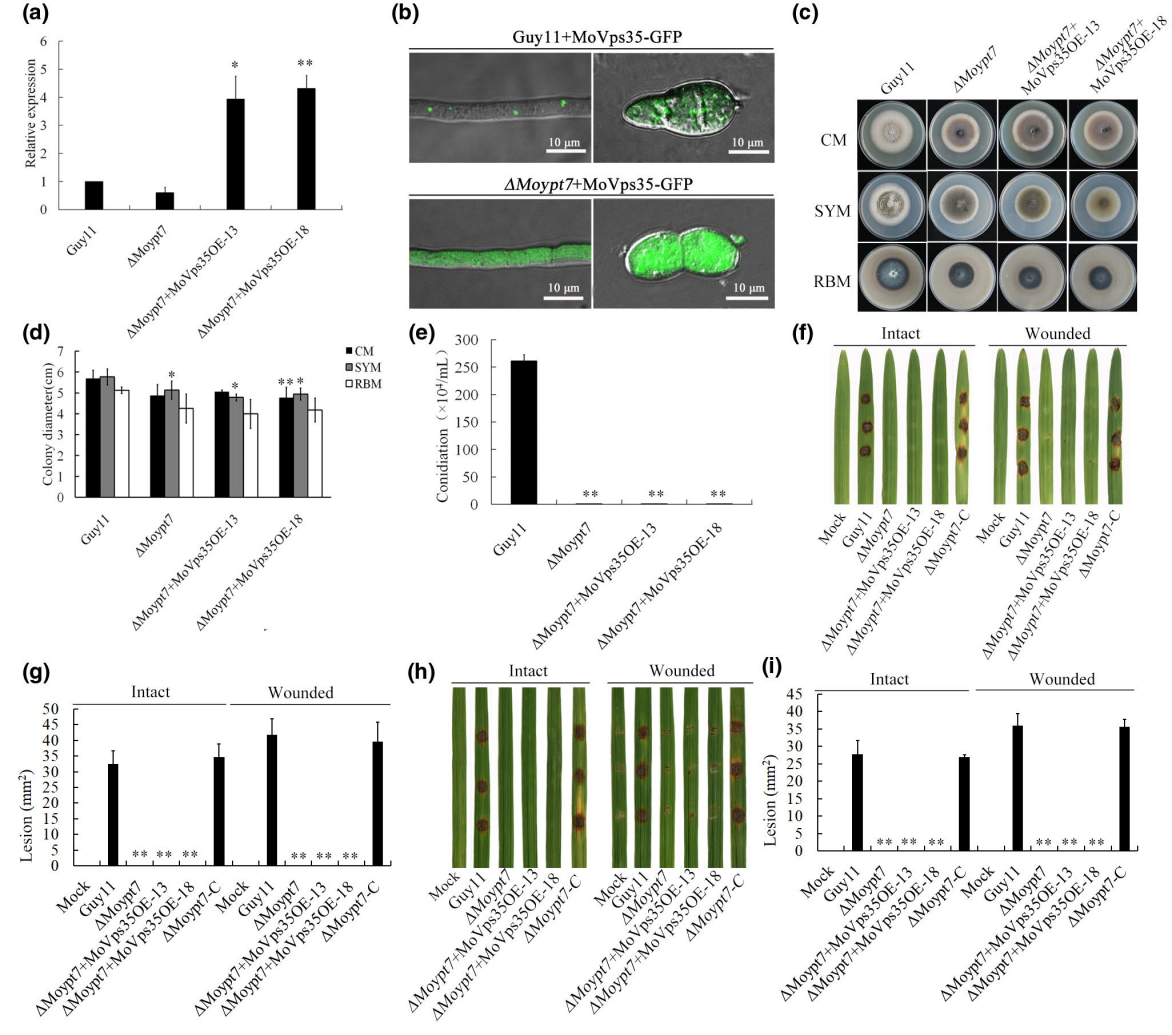
Overexpression of *MoVps35* in Δ*Moypt7* failed to restore the phenotypic defects observed in Δ*Moypt7*. (a) The relative expression level of *MoVps35* in the indicated strains. Standard deviations were calculated from three biological replicates. Level of significance was measured using an unpaired *t* test (*p < .05, **p < .01). (b) MoVps35OE‐GFP is distributed in cytoplasm of the mycelia and conidia of Δ*Moypt7*. Bar = 10 µm. (c) Mycelial growth of Guy11, Δ*Moypt7* mutant and two independent *MoVps35* overexpression transformants in the Δ*Moypt7* background on complete medium (CM), starch yeast medium (SYM), and rice bran medium (RBM) after 10 days. (d) Analysis of the colony diameters of the strains in Figure 5c. Level of significance was measured using an unpaired *t* test (*p < .05, **p < .01). (e) Comparison of the conidiation ability of the various strains on RBM. Strains were cultured on RBM at 28 °C and then the hyphae were stripped out and reincubated until new aerial hyphae filled up the plates. After 4 days of incubation with a 12 hr photoperiod, conidia were harvested and quantified using haemocytometer. The level of significance was measured using an unpaired *t* test (**p < .01). (f) Disease symptoms on intact and wounded barley leaves infected with mycelial blocks of the wild type (Guy11), Δ*Moypt7* mutant (Δ*Moypt7*), *MoVps35* overexpression transformants (Δ*Moypt7 + MoVps35*OE‐13 and Δ*Moypt7 + MoVps35*OE‐18) and the complemented strain of Δ*Moypt7* (Δ*Moypt7*‐C). (g) Quantification of lesion area from the inoculated barley leaves. The level of significance was measured using an unpaired *t* test (**p < .01, *n* = 9 lesions, error bars indicate standard deviation). (h) Disease symptoms on intact and wounded rice leaves infected with mycelial blocks of the wild type (Guy11), Δ*Moypt7* mutant (Δ*Moypt7*), *MoVps35* overexpression transformants (Δ*Moypt7 + MoVps35*OE‐13 and Δ*Moypt7 + MoVps35*OE‐18), and the complemented strain of Δ*Moypt7* (Δ*Moypt7*‐C). (i) Quantification of lesion area from the inoculated rice leaves. The level of significance was measured using an unpaired *t* test (**p < .01, *n* = 9 lesions, error bars indicate standard deviation)

To determine whether an increase in the expression level of *MoVps35* could rescue the phenotypic defects of the Δ*Moypt7* mutant, we overexpressed *MoVps35* in the Δ*Moypt7* mutant and found that the expression levels of *MoVps35* in two transformants, Δ*Moypt7 + MoVps35*OE‐13 and Δ*Moypt7 + MoVps35*OE‐18, were 3.93‐ and 4.31‐fold up‐regulated compared to the expression level of *MoVps35* in Guy11, respectively (Figure 4a). The overexpressed MoVps35‐GFP was mainly dispersed in the cytoplasm in the mycelia and conidia of the Δ*Moypt7* mutant (Figure 4b). This is consistent with the expression of MoVps35‐GFP under the native *MoVps35* promoter in the Δ*Moypt7* mutant (Figure 2a). Furthermore, we compared the vegetative growth rates of Guy11, Δ*Moypt7*, Δ*Moypt7 + MoVps35*OE‐13, and Δ*Moypt7 + MoVps35*OE‐18 on complete medium (CM), starch yeast medium (SYM), and rice bran medium (RBM) in hyphal growth assays. The vegetative growth rates of Δ*Moypt7 + MoVps35*OE‐13 and Δ*Moypt7 + MoVps35*OE‐18 were not significantly different from that of Δ*Moypt7* on CM, SYM, and RBM (Figure 4c,d). The conidiation defects of the overexpressed strains remained the same as the Δ*Moypt7* mutant (Figure 4e). In pathogenicity tests, all the *MoVps5*‐overexpressing strains exhibited a similar degree of pathogenicity to the Δ*Moypt7* mutant (Figure 4f–i). Thus, our results showed that the overexpression of *MoVps35* in Δ*Moypt7* did not restore the phenotypic defects of Δ*Moypt7* to the wild‐type phenotype. We also performed a reciprocal experiment by overexpressing *MoYpt7* in the Δ*Movps35* mutant. The results were the same (Figure S3), suggesting that the regulatory relationship between MoYpt7 and MoVps35 is not decided by transcriptional control but depends on their accurate spatial positioning in the cells.

### 2.6 MoVps17 releases MoVps35 after its recruitment to the endosomal membrane by MoYpt7

**FIGURE 5:**
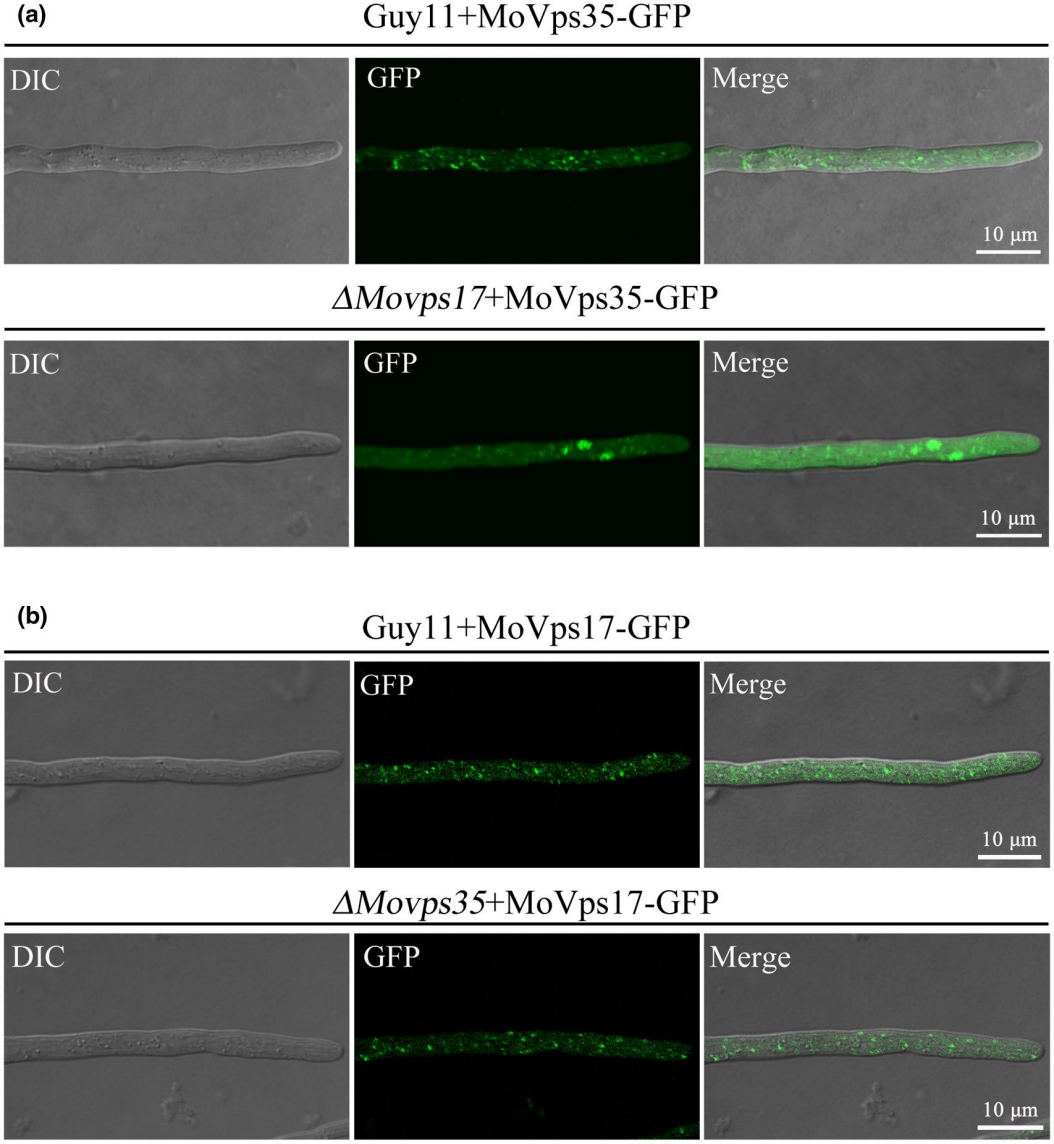
Differential interference constrast (DIC) and fluorescent images of MoVps35‐GFP expressed in the Δ*Movps17* mutant. (a) Comparison of the localization of MoVps35‐GFP in the wild type and the Δ*Movps17* mutant. MoVps35‐GFP was significantly aggregated in Δ*Movps17* hyphae, indicating that MoVps17 is required for MoVps35 fission. (b) Comparison of the localization of MoVps17‐GFP in the wild type and Δ*Movps35* mutant. Deletion of *MoVps35* does not impair the punctate localization of MoVps17. Bar = 10 μm

The precise sorting event of the retromer CSC after recruitment to the endosomal membrane and any potential cross‐regulation among the retromer CSC, SNX, and Ypt7 have not been investigated in depth, even in mammals and yeast. To our knowledge, this process is also completely uninvestigated in filamentous fungi. To investigate this gap, we analysed the role of the sorting nexin (SNX) dimer MoVps17/MoVps5 with respect to MoVps35 and MoYpt7. Our previous study has already established the essentiality of MoVps5 in *M. oryzae* development (Zheng et al., 2017). As such, we focus here on the role of MoVps17 in relation to these proteins. We first investigated the localization of MoVps35‐GFP in the Δ*Movps17* mutant. We found that MoVps35‐GFP was aggregated into plaque‐like structures and there was significant reduction of discrete fluorescent spots compared to the wild type (Figure 5a). This suggests that MoVps17 regulates the sorting and trafficking function of MoVps35 after its recruitment by MoYpt7. To determine whether MoVps35 could regulate the localization MoVps17, we transformed *MoVps17‐GFP* into the Δ*Movps35* mutant and the results showed that the localization of MoVps17‐GFP was not affected in the Δ*Movps35* mutant compared to the wild type (Figure 5b). Therefore, our data show that the endosomal membrane localization of the sorting nexin protein MoVps17 does not depend on retromer CSC but plays an important role in separating/fission retromer CSC.

**FIGURE 6:**
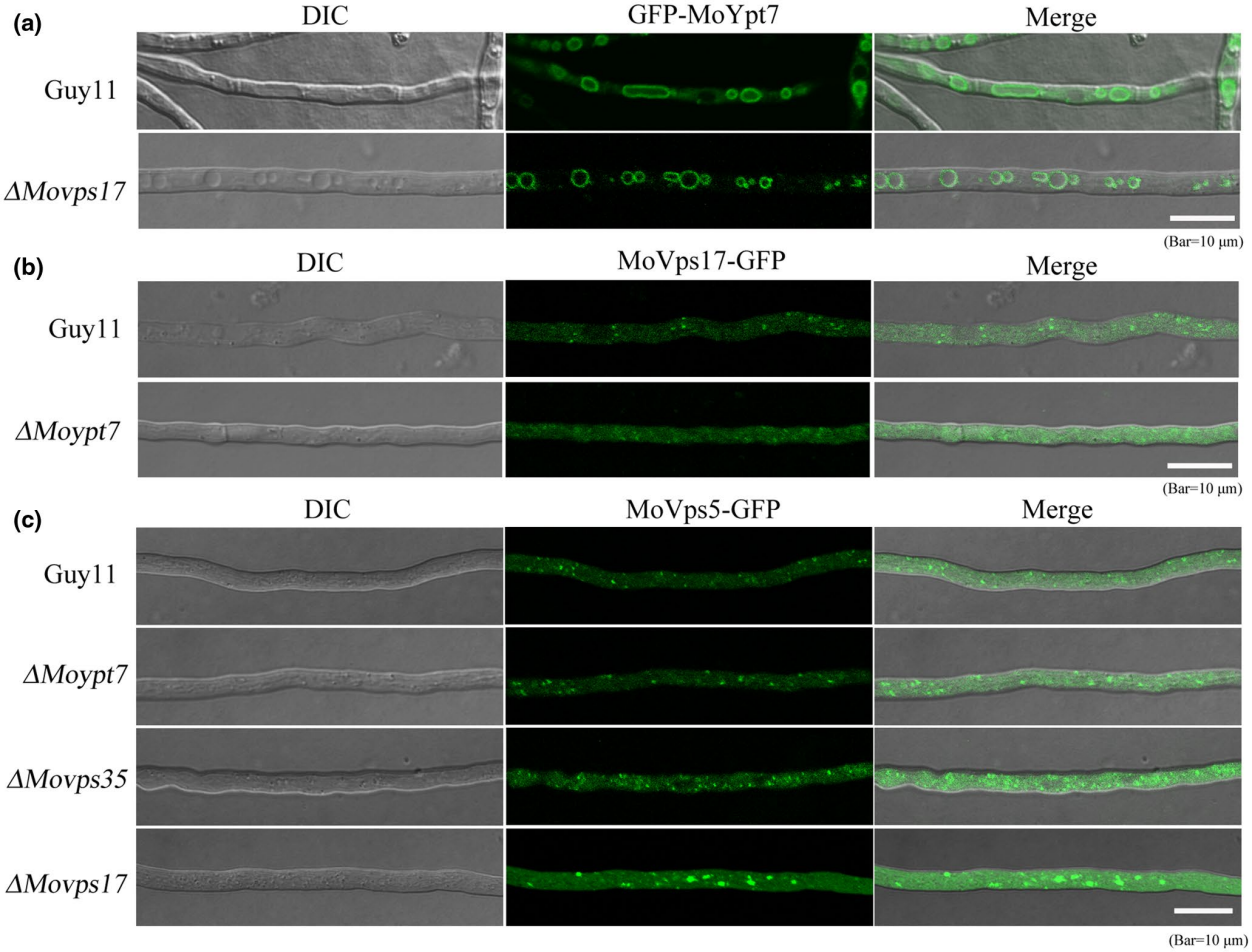
Relationship among MoYpt7, MoVps35, and MoVps17 in *Magnaporthe oryzae*. (a) Localization of GFP‐MoYpt7 in the vegetative hyphae of the wild type Guy11 and Δ*Movps17* mutant. Deletion of *MoVps17* does not impair the vacuolar membrane localization of MoYpt7. (b) Localization of MoVps17‐GFP in the vegetative hyphae of Guy11 and Δ*Moypt7*. Loss of MoYpt7 affects the punctate pattern of MoVps17‐GFP. (c) Localization of MoVps5‐GFP in the vegetative hyphae of Guy11, Δ*Moypt7*, Δ*Movps35*, and Δ*Movps17*. MoVps5‐GFP was significantly aggregated in Δ*Movps17* mutant hyphae, indicating that MoVps17 is required for MoVps5 fission. Bar = 10 μm

We examined if there is any connection between the MoVps35 recruiting protein MoYpt7 and MoVps17. To this end, we determined the localization of GFP‐MoYpt7 in the Δ*Movps17* mutant and vice versa. Our results indicated that MoVps17 was dispensable for normal endosomal/vacuolar membrane localization of MoYpt7 (Figure 6a). However, deletion of *MoYpt7* partially disrupted the punctate localization of MoVps17 (Figure 6b). Finally, we investigated the localization of the second SNX component of the retromer complex MoVps5 in Δ*Moypt7,* Δ*Movps35*, and Δ*Movps17* mutants. The results demonstrated that the localization of MoVps5 was not affected in Δ*Moypt7* and Δ*Movps35*. However, some MoVps5‐GFP aggregates were observed in Δ*Movps17* (Figure 6c), suggesting that MoVps17 is involved in the sorting of MoVps5 in *M. oryzae*. These results suggest that consecutive recruitment and sorting of retromer CSC is carried out by MoYpt7 and MoVps17, respectively, which is crucial to coordinate material/protein delivery for efficient conidiation and pathogenicity in pathogenic fungi.

### 2.7 Δ***Moypt7* and** Δ***Movps17* mutants show more severe pathogenicity defects than the retromer CSC mutants**

**FIGURE 7:**
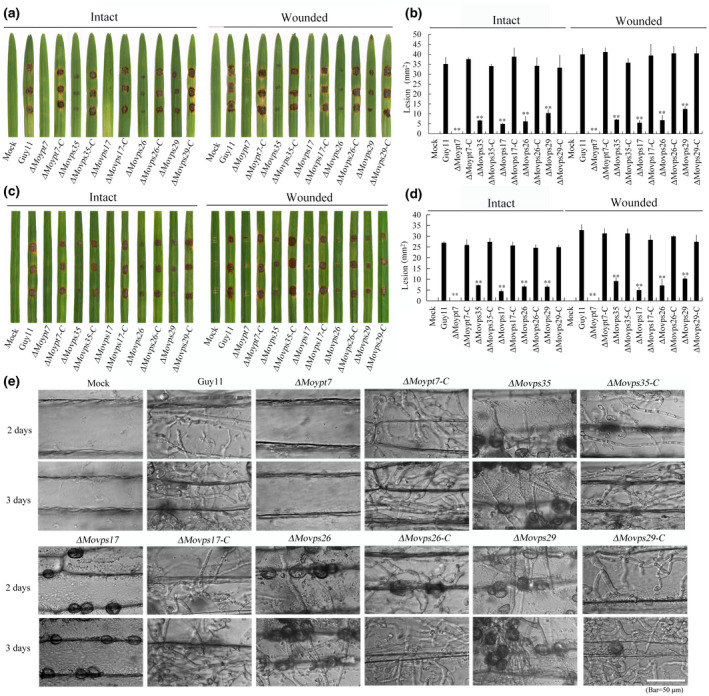
The Rab GTPase MoYpt7 and the retromer complex are important for pathogenicity in *Magnaporthe oryzae*. (a) Disease symptoms due to mycelial blocks from wild type (Guy11), Δ*Moypt7*, Δ*Moypt7*‐C, Δ*Movps17*, Δ*Movps17*‐C, Δ*Movps35*, Δ*Movps35*‐C, Δ*Movps26*, Δ*Movps26*‐C, Δ*Movps29*, and Δ*Movps29*‐C strains, inoculated on intact and wounded barley leaves for 7 days. Compared to the wild type and each complemented (‐C) strain, Δ*Movps35*, Δ*Movps26*, and Δ*Movps29* are significantly reduced in virulence while Δ*Moypt7* and Δ*Movps17* almost lost their pathogenicity on the host. (b) Quantification of lesion area from the inoculated barley leaves. The level of significance was measured using an unpaired *t* test (**p < .01, *n* = 9 lesions, error bars indicate standard deviation).(c) Disease symptoms due to mycelial blocks from wild type, Δ*Moypt7*, Δ*Moypt7*‐C, Δ*Movps17*, Δ*Movps17*‐C, Δ*Movps35*, Δ*Movps35*‐C, Δ*Movps26*, Δ*Movps26*‐C, Δ*Movps29*, and Δ*Movps29*‐C strains, inoculated on intact and wounded rice leaves for 7 days. Compared to the wild type and each complemented strain, Δ*Movps35*, Δ*Movps26*, and Δ*Movps29* are significantly reduced in virulence while Δ*Moypt7* and Δ*Movps17* almost lost their pathogenicity on the host. (d) Quantification of lesion area from the inoculated rice leaves. The level of significance was measured using an unpaired *t* test (**p < .01, *n* = 9 lesions, error bars indicate standard deviation).(e) Microscopic observations for penetration of wild type, Δ*Moypt7*, Δ*Moypt7*‐C, Δ*Movps17*, Δ*Movps17*‐C, Δ*Movps35*, Δ*Movps35*‐C, Δ*Movps26*, Δ*Movps26*‐C, Δ*Movps29*, and Δ*Movps29*‐C mycelia inoculated on barley leaves for 2 and 3 days. Extensive hyphae are found in the barley epidermal cells when inoculated with the wild type and each of the complemented strains. Δ*Movps35*, Δ*Movps26*, and Δ*Movps29* can also penetrate the barley epidermal cells and produce invasive hyphae, while the hyphal growth of Δ*Moypt7* and Δ*Movps17* ceased completely. Bar = 50 µm

To compare the roles of the Rab GTPase MoYpt7 and the components of the retromer CSC (MoVps26, MoVps29, and MoVps35) and the sorting nexin protein MoVps17 in *M. oryzae* pathogenicity, we cultured their respective deletion mutants and complemented strains on CM. Subsequently, each strain was inoculated on both intact and wounded barley as well as rice leaves for infection assays. Compared to the wild‐type strain (Guy11) and each complemented strain, the pathogenicity of *MoYpt7*, *MoVps17*, *MoVps35*, *MoVps26*, or *MoVps29* gene deletion mutants were either significantly reduced or completely abolished (Figure 7a–d). For more detailed analyses, we further investigated their penetration and host cell colonization abilities in epidermal cells of barley leaves. Our results showed that the Δ*Moypt7* and Δ*Movps17* mutants were almost completely absent from the host epidermal cells, whereas the deletion mutants of the retromer CSC components showed some levels of colonization that was less than that of the wild type (Figure 7e). Taken together, our results demonstrate that MoYpt7 and all the retromer complex subunits are necessary for pathogenicity of *M. oryzae,* and that Δ*Moypt7* and Δ*Movps17* mutants appear less pathogenic than the mutants of the retromer CSC subunits.

## 3 DISCUSSION

**FIGURE 8:**
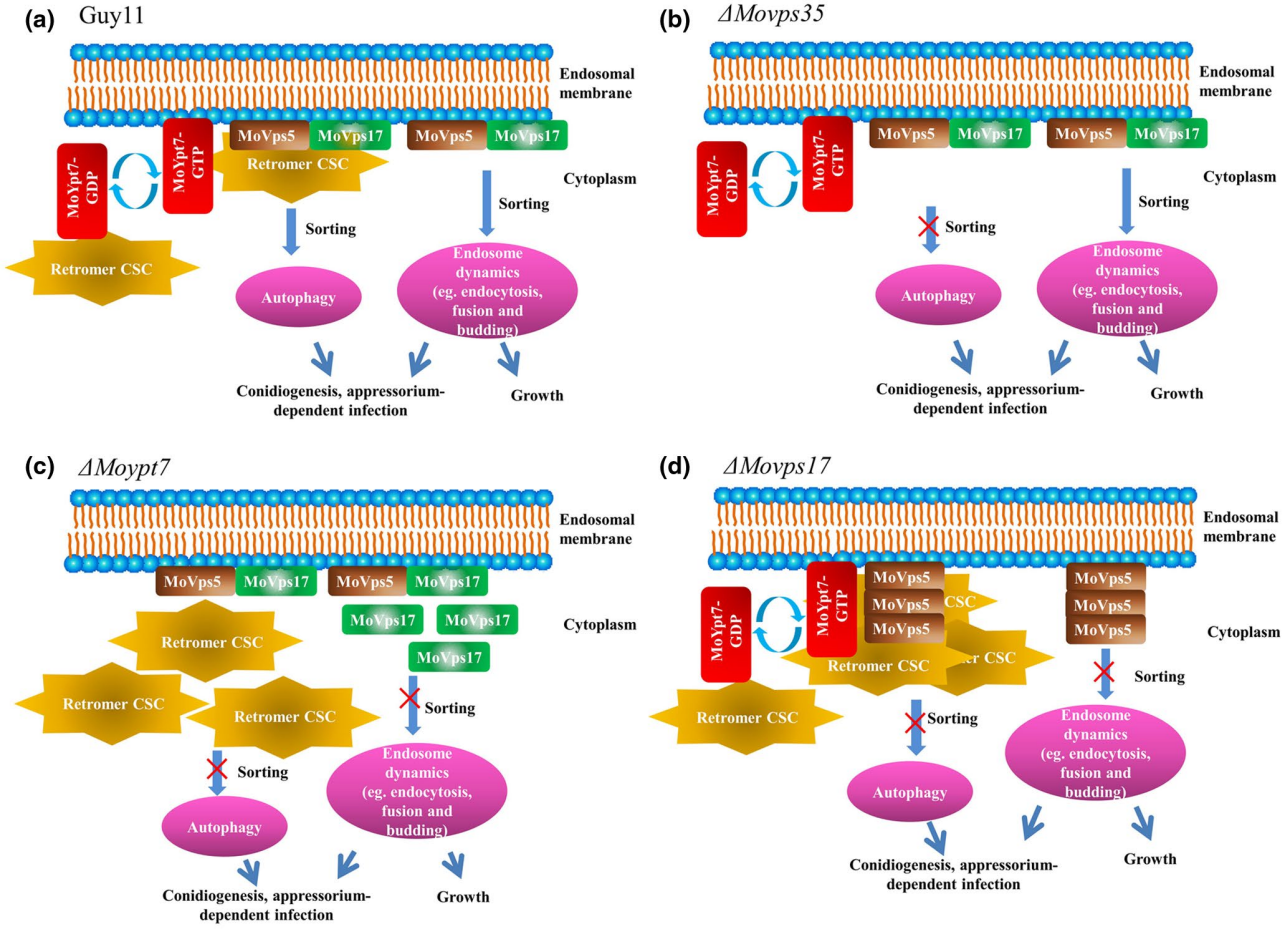
A proposed model depicting the relationship between MoYpt7 and retromer and their regulation mechanisms in *Magnaporthe oryzae* development and pathogenesis. (a) MoYpt7 interacts with MoVps35 and this interaction is independent of whether MoYpt7 is in the activated (GTP‐bound) or inactivated (GDP‐bound) state. MoVps35 is recruited to the vacuolar membrane by MoYpt7 to mediate protein trafficking, and then separated from MoYpt7 by MoVps17. The sorting functions of MoVps35 and MoVps17 are important for autophagy and endosome dynamics, respectively, which mediate the effective conidiation and pathogenicity of *M. oryzae*. (b) Deletion of MoVps35 does not alter the functions of MoYpt7 and MoVps17 but attenuates conidiation and pathogenicity of *M. oryzae* through perturbation of autophagy. (c) Deletion of *MoYpt7* results in mislocalization of MoVps35 and MoVps17 to the cytoplasm, which leads to defective autophagy and endosome dynamics. (d) MoVps17 regulates the sorting function of MoVps35 after being recruited by MoYpt7. MoVps35 and MoVps5 are significantly aggregated in the Δ*Movps17* mutant, signifying that the sorting function of MoVps35 is blocked due to deletion of MoVps17

In this study, we investigated the molecular mechanism of the indispensable vesicular transport machinery, the retromer CSC, in the phytopathogenic fungus *M. oryzae*. To summarize our findings, the model in Figure 8a is proposed, in which MoYpt7 initially recruits the retromer CSC from the cytosol to the endosomal membrane. After this recruitment, MoVps17 subsequently replaces MoYpt7 and assembles with the retromer CSC to mediate protein sorting/trafficking. In *M. oryzae*, protein sorting is essential for inducing autophagy, which is required for effective conidiation and pathogenicity (Abubakar et al., 2017; Zheng et al., 2015b). In addition, the sorting nexin proteins MoVps17/MoVps5 play distinct roles in the regulation of endosome dynamics during fungal development and plant infection, which was documented in our previous study (Zheng et al., 2017). Notably, deletion of *MoVps35* does not influence the subcellular localization of MoYpt7, MoVps5, and MoVps17, but the autophagy‐dependent conidiation and pathogenicity of *M. oryzae* ceases (Figure 8b) (Abubakar et al., 2017; Zheng et al., 2015b). In the absence of MoYpt7, the retromer CSC and MoVps17 are mislocalized to the cytoplasm, which severely affects autophagy and the endosome dynamics‐dependent conidiation and pathogenicity of *M. oryzae* (Figure 8c). In the Δ*Movps17* mutant, the balance between recruitment and sorting is perturbed, which results in excessive accumulation of CSC and MoVps5 on the endosomal membrane (Figure 8d). In conclusion, our findings provide the precise mechanism of recruitment of the retromer CSC subcomplex as well as of its sorting function, which are essential for development and pathogenicity of *M. oryzae*.

*MoYpt7* and the retromer complex components *MoVps17*, *MoVps35*, *MoVps26*, and *MoVps29* have been reported to play vital roles in the regulation of pathogenicity in *M. oryzae* (Liu et al., 2015; Zheng et al., 2015b, 2017). The present study confirmed that the pathogenicity of Δ*Movps35*, Δ*Movps26*, and Δ*Movps29* mutants were significantly reduced compared to the wild‐type strain, while Δ*Moypt7* and Δ*Movps17* mutants almost lost their pathogenicity in the host (Figure 7), indicating that MoYpt7 and MoVps17 play stronger regulatory roles in pathogenicity than the retromer CSC complex in *M. oryzae*. Our previous study showed that the SNX protein MoVps17 localizes to endosomes and is essential for regulating endosome dynamics such as endosome budding, fusion, and endocytosis, which are independent of the retromer CSC (Zheng et al., 2017), meaning that the tethering of MoVps17 to the endosomal membrane further strengthens the ability of the pathogen to infect the host plant. MoYpt7 acts as a molecular switch for vesicle transport where deletion of the *MoYpt7* gene prevents the transition from early to late endosomes or vacuole/lysosomes, which leads to defects in multiple regulatory pathways and autophagy (Liu et al., 2015). This explains why the Δ*Moypt7* mutant in this study almost lost the ability to infect the host.

Endosomes are important protein‐sorting centres during endocytosis in eukaryotes (Huotari & Helenius, 2011). The cargoes of the endocytic system could be retained in mature endosomes and delivered to lysosomes for degradation, or could be transported to other organelles (Seaman et al., 1998). The retromer complex prevents unnecessary degradation of cargoes by sorting them from endosomes and channelling them to the Golgi network via the retrograde trafficking pathway (Attar & Cullen, 2010; Rojas et al., 2008; Seaman et al., 1998). In mammals, Rab7 interacts with the retromer CSC complex Vps26‐Vps29‐Vps35. A pull‐down assay indicated that Rab7 colocalizes with retromer complex on the endosomes, and the retromer CSC complex Vps26‐Vps29‐Vps35 is recruited to the endosomal membrane by GTP‐bound Rab7 protein (Priya et al., 2015; Rojas et al., 2008). In *Arabidopsis*, the Rab7 homolog RABG3f plays a role in recruiting the retromer CSC to the endosomal membrane through interaction with Vps35 (Zelazny et al., 2013). This recruitment requires Ypt7, a protein that coordinates retromer‐mediated retrograde trafficking, membrane recycling, and fusion of late endosomes with the vacuole in *S. cerevisiae* (Balderhaar et al., 2010; Harrison et al., 2014; Liu et al., 2012; Purushothaman et al., 2017). In this study, our pull‐down and mass spectrometry analyses indicated that MoYpt7 interacts with MoVps35 (Table 1), which has been confirmed by Co‐IP experiments (Figure 1d). mCherry‐MoYpt7 colocalizes with MoVps35‐GFP on late endosomes/vacuolar membrane (Figure 1a,b), which is consistent with previous studies in other species (Balderhaar et al., 2010; Harrison et al., 2014; Liu et al., 2012; Purushothaman et al., 2017; Rojas et al., 2008; Zelazny et al., 2013). These results suggest that the interaction of Ypt7 with Vps35 is conserved among different species.

Rab proteins regulate various pathways by interacting with some effector proteins. In yeast, previous studies on the retromer complexes showed that the retromer CSC interacts with Ypt7‐GTP (Balderhaar et al., 2010; Liu et al., 2012; Purushothaman et al., 2017). Our results showed that both the GTP‐bound (MoYpt7‐CA) and GDP‐bound (MoYpt7‐DN) forms of MoYpt7 interacted with MoVps35 (Figure 3c), which means that this interaction is independent of the activate or inactivate state of MoYpt7. Our finding is consistent with a study from mammalian cells that showed that the dissociation constants for the interactions of Vps35 with GppNHp‐ and GDP‐bound Rab7 were similar, indicating Vps35 does not show any preference for the active form of Rab7 (Priya et al., 2015). Most effector proteins interact with Rab proteins in their activate states, but there are a few effector proteins that interact with inactivate Rab proteins (Shirane & Nakayama, 2006). Therefore, the MoVps35 may not act as a typical effector of MoYpt7 in *M. oryzae*. This clearly demonstrates a difference in the regulation of retromer function between yeast and filamentous fungi.

We also revealed that the *M. oryzae* retromer exhibited unexpected features compared to its yeast counterparts. For example, we found that MoVps35‐GFP fluorescence was aggregated into plaque‐like structures and the discrete fluorescent spots were significantly reduced in the Δ*Movps17* mutant compared to the wild type (Guy11) (Figure 5). In yeast, although the localization of retromer CSC to endosomes requires the Vps5 and Vps17 sorting nexins, it does not show aggregated structures in the Δ*vps5vps17* double mutant (Liu et al., 2012). In addition, loss of Ypt7 does not lead to mislocalization of Vps35‐GFP to the cytoplasm in the yeast (Liu et al., 2012). A further study found that yeast Vps35‐GFP and Vps26‐GFP localize to the cytosol in a Δ*vps5vps17ypt7* triple mutant (Liu et al., 2012). These data indicate that the *M. oryzae* sorting nexin MoVps17 itself plays a vital role in retromer CSC sorting. We also investigated the connection between MoYpt7 and MoVps17 and found that the punctate localization of MoVps17 was partially affected in a *MoYpt7* mutant, and MoVps17 was not required for endosomal/vacuolar membrane localization of MoYpt7 (Figure 6a,b). The punctate localization of MoVps5 was not affected in the *MoYpt7* and *MoVps35* mutants, but disruption of *MoVps17* resulted in MoVps5 accumulation into plaque‐like structures (Figure 6c). These results further confirm that MoVps17 plays a leading role in vesicular sorting, which is clearly different from its role in yeast.

Overexpression of *MoYpt7* in Δ*Movps35* did not restore the phenotypic defects of Δ*Movps35* (Figure S3). Likewise, overexpression of *MoVps35* in Δ*Moypt7* did not restore the phenotypic defects observed in Δ*Moypt7* (Figure 4). These results indicate that the biological function in conidogenesis and pathogenicity depends on correct recognition of the retromer by MoYpt7 at the right time and in the right space. Therefore, our results fill the gap in knowledge between retromer complex recruitment and its sorting function in filamentous fungi, which could lay a foundation for effective prevention and control of rice blast disease.

## 4 EXPERIMENTAL PROCEDURES

### 4.1 Fungal strains, media, and growth conditions

Guy11 and Ku70 were used as the wild‐type strains from which the deletion mutants Δ*Moypt7*, Δ*Movps35*, and other mutant strains were generated (Table S2). The various strains were cultured at 28 °C with a 12 hr photoperiod. The media used in the assays included CM (Zheng et al., 2015b), SYM (0.3% sucrose, 1% starch, 0.2% yeast extract, 2% agar), and RBM (4% rice bran, pH 6.0–6.5, 2% agar).

### 4.2 Construction of fusion vectors and point mutations

MoYpt7 was tagged at the N‐terminus with mCherry, with the gene under the control of its native promoter, to generate the native‐mCherry‐MoYpt7 vectors. This insertion was verified by PCR using the specific primer pairs presented in Table S1. The mCherry fragment was amplified from the FgSpa2‐mCherry plasmid (Zheng et al., 2015a) and then cloned into the pCB1532 plasmid digested with *Sal*I and *Bam*HI. The MoYpt7 protein was also tagged with GFP at its N‐terminus, with the gene under the control of native and RP27 promoters. The *GFP* fragment was isolated from the GFP‐FgRab fusion vector (Zheng et al., 2015a), whereas the RP27 fragment was isolated from the pTE11 vector and cloned into plasmid pKNT following digestion with *Xho*I and *Hin*dIII restriction enzymes. For generation of MoYpt7‐CA and MoYpt7‐DN constructs, we first amplified *MoYpt7‐* *CA1*, ‐*CA2*, ‐*DN1*, and ‐*DN2* genes from the cDNA of Guy11 using the pairs of primers presented in Table S1. We then introduced the point mutations in *MoYpt7‐* *CA* and *MoYpt7‐* *DN* fragments using splicing‐by‐overlap extension PCR and cloned them (with an RP27 promoter) into pKNT vector and verified the constructs by sequencing. MoVps35 was also tagged with GFP at its C‐terminus, with the gene under the control of its native or RP27 promoter, to form the native‐MoVps35‐GFP or RP27‐MoVps35‐GFP vectors. The constructed vectors were transformed into Guy11, Δ*Moypt7*, or Δ*Movps35* mutant protoplasts.

### 4.3 Co‐IP assay

We coexpressed MoYpt7 fused with GFP and MoVps35 tagged with FLAG in Guy11 protoplasts. The total proteins were extracted from the transformants and GFP‐Trap beads (ChromoTek Inc.) were used for a pull‐down assay. The interacting proteins were separated by SDS‐PAGE. The GFP‐MoYpt7 and the MoVps35‐FLAG proteins were detected by α‐GFP (MBL) and α‐FLAG (Abmart) antibodies, respectively.

### 4.4 Quantitative reverse transcription PCR

Total RNA was extracted from the wild type and mutants’ mycelia harvested from liquid CM that was inoculated and incubated at 28 °C with constant shaking at 110 rpm for 3 days, using an Eastep Super Total RNA Extraction Kit (Promega). Reverse transcription was used to generate cDNA using a reverse transcription kit (Takara). Quantitative PCR was finally conducted using a SYBR kit (Takara) with specific primers (Table S1). *M. oryzae* *β‐tubulin* (Table S1) was used as the endogenous reference gene, and the expression levels were calculated using the 2^−ΔΔ^ *^C^* ^t^ method as previously reported (Livak & Schmittgen, 2001). All RT‐qPCR assays were repeated three times for each sample.

### 4.5 Subcellular localization assay

For cellular localization assay, all the strains were cultured in liquid/solid CM at 28 °C. The mycelia and conidia of the strains involved were observed using a Nikon A1R laser scanning confocal microscope. Unless noted otherwise, all microscope images were taken within the same focal plane section.

### 4.6 Phenotypic analysis

For growth rate assays, mycelial blocks 3 mm in diameter (the peripheral edge of the colony) were inoculated at the centre of CM, SYM, and RBM plates. We measured the diameters of the colonies after 10 days of incubation at 28 °C. For conidiation assays, the strains were cultured on RBM plates and incubated at 28 °C and the first aerial hyphae were stripped out after they had filled up the plates, and reincubated. After 4 days of the second incubation, the cultures were washed with double‐distilled water and conidia were collected and counted using a haemocytometer. For the pathogenicity assay, fresh mycelial blocks were inoculated on intact and wounded barley (cv. Golden Promise) as well as rice (CO39) leaves. The set‐up was kept humid and incubated in the dark for 24 hr at 28 °C, then transferred to a 12 hr photoperiod at 28 °C. The lesions were observed and compared after 7 days. The lesion area was analysed using ImageJ software.

## Supporting information


**FIGURE S1** Defects in vegetative growth, conidiation, and pathogenicity of Guy11 strain expressing MoYpt7‐DN. (a) The expression level of MoYPT7‐DN in Guy11. The level of significance was measured using an unpaired *t* test (*p < .05, **p < .01). (b) GFP‐MoYpt7‐DN is distributed within the cytoplasm in the mycelia and conidia of Guy11. (c)–(i) Mycelial growth (c, d), conidiation (e), and pathogenicity (f–i) of MoYpt7‐DN‐expressing strains. The level of significance was measured using an unpaired *t* test (*p < .05, **p < .01)

**FIGURE S2** Defects in vegetative growth, conidiation, and pathogenicity of Guy11 strain expressing MoYpt7‐CA. (a) The expression level of MoYpt7‐CA in Guy11. The level of significance was measured using an unpaired *t* test (*p < .05, **p < .01). (b) GFP‐MoYpt7‐CA is localized to the endosomal/vacuolar membrane in the mycelia and conidia of Guy11. (c)–(i) Vegetative growth (c, d), conidiation (e), and pathogenicity (f–i) of the MoYpt7‐CA‐expressing strains. The level of significance was measured using an unpaired *t* test (*p < .05, **p < .01)

**FIGURE S3** Overexpression of *MoYPT7* in Δ*Movps35* failed to restore the phenotypic defects observed in Δ*Movps35* mutant. (a) The relative expression levels of *MoYPT7* in each strain. Standard deviations were calculated from three biological replicates. Significance was measured using an unpaired *t* test (*p < .05, **p < .01). (b) GFP‐MoYpt7OE localized to vacuolar membranes in the mycelia and conidia of Δ*Movps35* mutant. Bar = 10 µm. (c)–(i) The vegetative growth (c, d), conidiation (e), and pathogenicity (f–i) of MoYpt7OE in Δ*Movps35* strains. The level of significance was measured using an unpaired *t* test (*p < .05, **p < .01)

**TABLE S1** Primers used in this study

**TABLE S2** Fungal strains used in this study

## Data Availability

The data that support the findings of this study are available from the corresponding author upon reasonable request.
