# Supplementary material for: The retromer CSC subcomplex is recruited by MoYpt7 and sequentially sorted by MoVps17 for effective conidiation and pathogenicity of the rice blast fungus
Source: Mol Plant Pathol. 2020 Dec 21;22(2):284–98. doi: 10.1111/mpp.13029 (PMC7814966; doi:10.1111/mpp.13029)
Supplement: Supplementary file 1 — FIGURE S1 Defects in vegetative growth, conidiation, and pathogenicity of Guy11 strain expressing MoYpt7‐DN. (a) The expression level of MoYPT7‐DN in Guy11. The level of significance was measured using an unpaired t test (*p < .05, **p < .01). (b) GFP‐MoYpt7‐DN is distributed within the cytoplasm in the mycelia and conidia of Guy11. (c)–(i) Mycelial growth (c, d), conidiation (e), and pathogenicity (f–i) of MoYpt7‐DN‐expressing strains. The level of significance was measured using an unpaired t test (*p < .05, **p < .01) [file MPP-22-284-s001.doc]

**
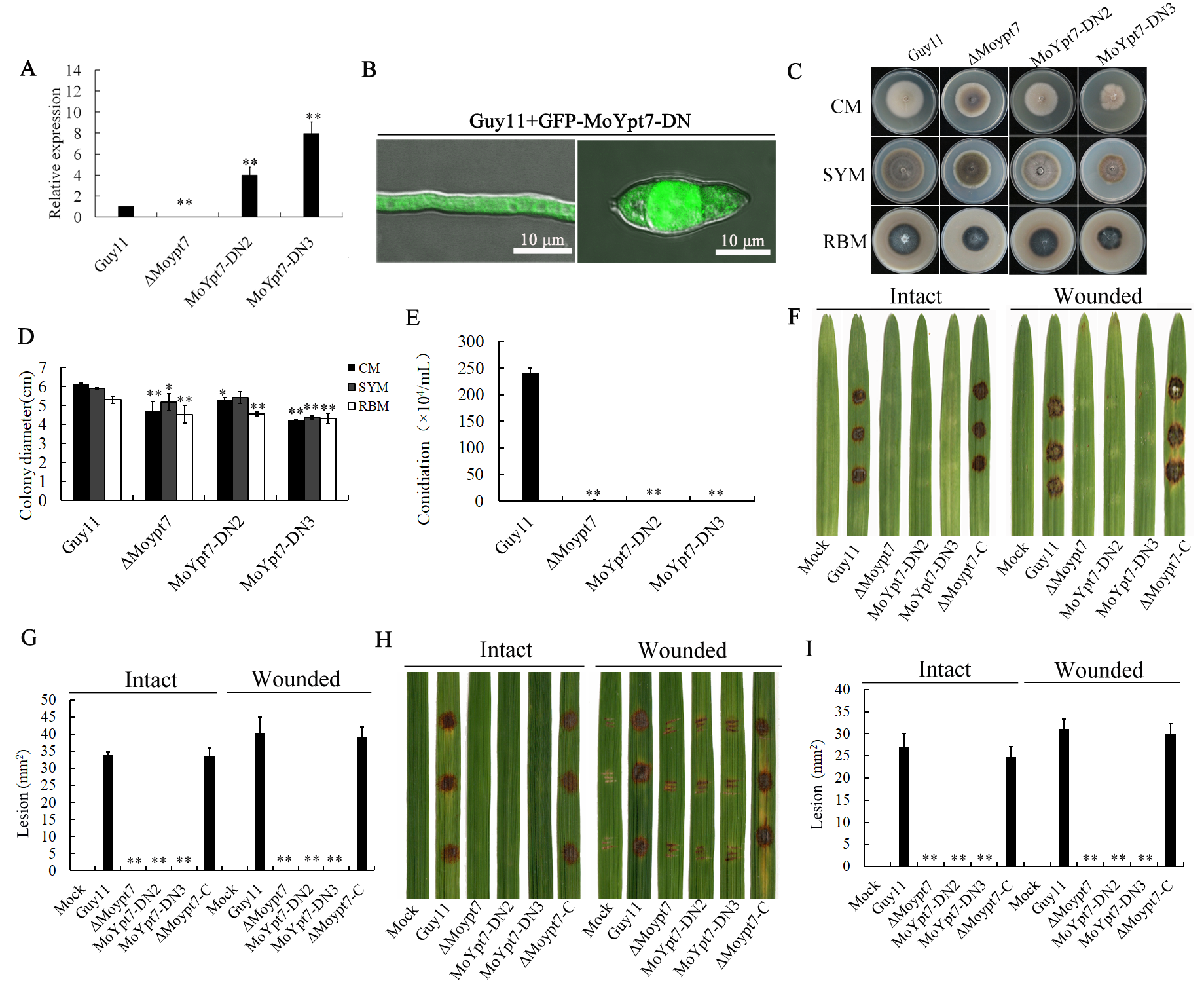
**

**Fig. S1 Defects in vegetative growth, conidiation and pathogenicity of Guy11 strain expressing MoYpt7-DN.**

(A) The expression level of MoYPT7-DN in Guy11. Level of significance was measured using unpaired t-test (* p<0.05, **p<0.01).

(B) GFP-MoYpt7-DN is distributed within the cytoplasm in the mycelia and conidia of Guy11.

(C-I) Mycelial growth (C.D), conidiation (E) and pathogenicity (F-I) of MoYpt7-DN-expressing strains. Level of significance was measured using unpaired t-test (* p<0.05, **p<0.01).
