# Supplementary material for: The retromer CSC subcomplex is recruited by MoYpt7 and sequentially sorted by MoVps17 for effective conidiation and pathogenicity of the rice blast fungus
Source: Mol Plant Pathol. 2020 Dec 21;22(2):284–98. doi: 10.1111/mpp.13029 (PMC7814966; doi:10.1111/mpp.13029)
Supplement: Supplementary file 2 — FIGURE S2 Defects in vegetative growth, conidiation, and pathogenicity of Guy11 strain expressing MoYpt7‐CA. (a) The expression level of MoYpt7‐CA in Guy11. The level of significance was measured using an unpaired t test (*p < .05, **p < .01). (b) GFP‐MoYpt7‐CA is localized to the endosomal/vacuolar membrane in the mycelia and conidia of Guy11. (c)–(i) Vegetative growth (c, d), conidiation (e), and pathogenicity (f–i) of the MoYpt7‐CA‐expressing strains. The level of significance was measured using an unpaired t test (*p < .05, **p < .01) [file MPP-22-284-s002.doc]

**
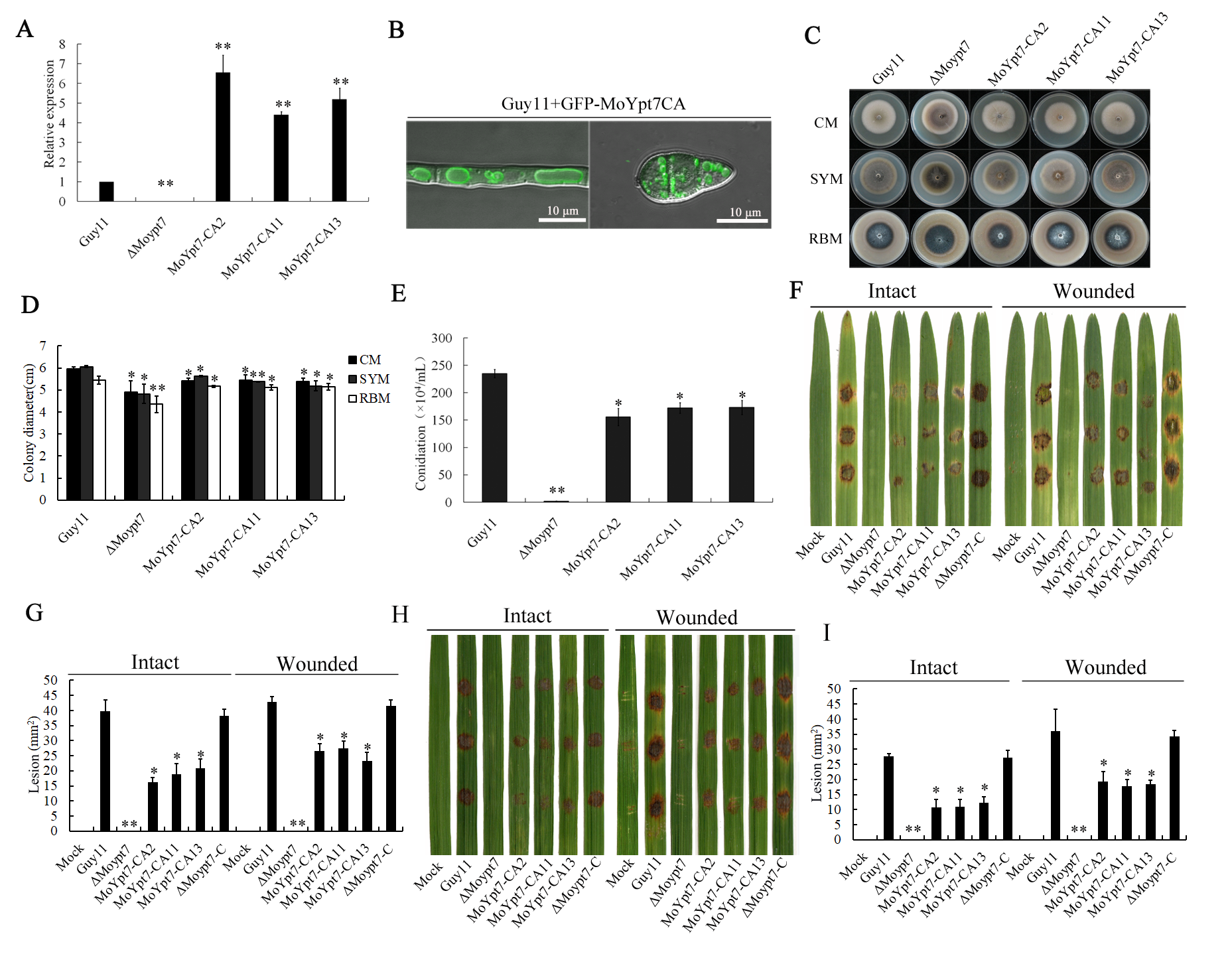
**

**Fig. S2 Defects in vegetative growth, conidiation and pathogenicity of Guy11 strain expressing MoYpt7-CA.**

(A) The expression level of MoYpt7-CA in Guy11. Level of significance was measured using unpaired t-test (* p<0.05, **p<0.01).

(B) GFP-MoYpt7-CA is localized to the endosomal/vacuolar membrane in the mycelia and conidia of Guy11.

(C-I) Vegetative growth (C.D), conidiation (E) and pathogenicity (F-I) of the MoYpt7-CA-expressing strains. Level of significance was measured using unpaired t-test (* p<0.05, **p<0.01).
