# Supplementary material for: The retromer CSC subcomplex is recruited by MoYpt7 and sequentially sorted by MoVps17 for effective conidiation and pathogenicity of the rice blast fungus
Source: Mol Plant Pathol. 2020 Dec 21;22(2):284–98. doi: 10.1111/mpp.13029 (PMC7814966; doi:10.1111/mpp.13029)
Supplement: Supplementary file 3 — FIGURE S3 Overexpression of MoYPT7 in ΔMovps35 failed to restore the phenotypic defects observed in ΔMovps35 mutant. (a) The relative expression levels of MoYPT7 in each strain. Standard deviations were calculated from three biological replicates. Significance was measured using an unpaired t test (*p < .05, **p < .01). (b) GFP‐MoYpt7OE localized to vacuolar membranes in the mycelia and conidia of ΔMovps35 mutant. Bar = 10 µm. (c)–(i) The vegetative growth (c, d), conidiation (e), and pathogenicity (f–i) of MoYpt7OE in ΔMovps35 strains. The level of significance was measured using an unpaired t test (*p < .05, **p < .01) [file MPP-22-284-s003.doc]

**
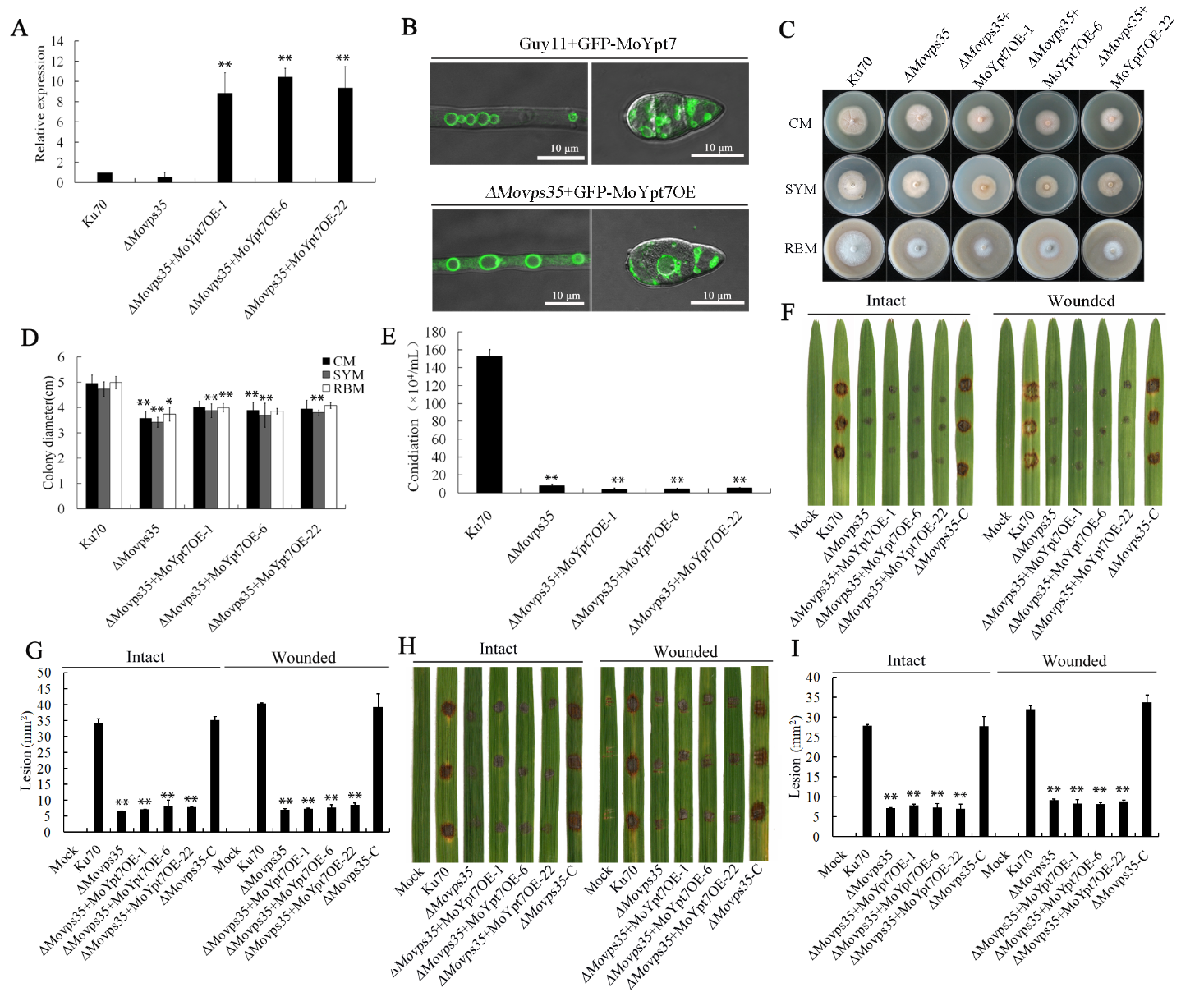
**

**Fig. S3 Overexpression of *MoYPT7* in *ΔMovps35* failed to restore the phenotypic defects observed in *ΔMovps35* mutant.**

(A) The relative expression levels of *MoYPT7* in each strain. Standard deviations were calculated from three biological replicates. Level of significance was measured using unpaired t-test (* p<0.05, **p<0.01).

(B) GFP-MoYpt7OE localized to vacuolar membranes in the mycelia and conidia of *ΔMovps35* mutant. Bars = 10 µm.

(C-I) The vegetative growth (C-D), conidiation (E) and pathogenicity (F-I) of MoYpt7OE in *ΔMovps35* strains. Level of significance was measured using unpaired t-test (* p<0.05, **p<0.01).
