# Supplementary material for: The retromer CSC subcomplex is recruited by MoYpt7 and sequentially sorted by MoVps17 for effective conidiation and pathogenicity of the rice blast fungus
Source: Mol Plant Pathol. 2020 Dec 21;22(2):284–98. doi: 10.1111/mpp.13029 (PMC7814966; doi:10.1111/mpp.13029)
Supplement: Supplementary file 4 — TABLE S1 Primers used in this study [file MPP-22-284-s004.doc]

**Table S1 primers used in this study.**

| Primers | Sequence 5’-3’ | Annotation |
| --- | --- | --- |
| MGG_08144PF1 | TGGCGGCCGCTCTAGAACTAGTTCCAGGTTTACGAAGCCAT | Generation of pMoRab7::mCherry-MoYpt7 vector（MGG_08144OF was used to construct pMoRab7::GFP-MoYpt7 and pRP27::GFP-MoYpt7OE vector） |
| MGG_08144PR1 | CTCGCCCTTGCTCACCATGTTGGGCGAGATGTTGTATC |
| mCherryF | ATGGTGAGCAAGGGCGAG |
| mCherryR | CTTGTACAGCTCGTCCAT |
| MGG_08144OF | GCATGGACGAGCTGTACAAGATGTCGTCCAGAAAGAAGGT |
| MGG_08144OR1 | GGTACCGGGCCCCCCCTCGAGTGAACTTAGTTTGGTGCTGGTA |
| MGG_08144PF2 | GGGTACCGGGCCCCCCCTCGAGTCCAGGTTTACGAAGCCAT | Generation of pMoRab7::GFP-MoYpt7 vector（MoYpt7-OF2 and MoYpt7-OR2 were used to construct pRP27::GFP-MoYpt7OE vector） |
| MGG_08144PR2 | GCTCCTCGCCCTTGCTCACCATGTTGGGCGAGATGTTGTATC |
| GFPF | ATGGTGAGCAAGGGCGAGGAGC |
| GFPR1 | CTTGTACAGCTCGTCCATGC |
| MGG_08144OR2 | CGACCTGCAGGCATGCAAGCTTTGAACTTAGTTTGGTGCTGGTA |
| RP27F | GGGTACCGGGCCCCCCCTCGAGGCCAGGGTTTTCCCAGTCA | Generation of pRP27::GFP-MoYpt7-CA and pRP27::GFP-MoYpt7-DN vectors（RP27F is a primer for all RP27 promoters in this study） |
| RP27R | GCTCCTCGCCCTTGCTCACCATTTTGAAGATTGGGTTCCTACG |
| MGG-08144CA1R | CTGAAATCTCTCCAGACCAGCAGTATC |
| MGG-08144CA2F | GATACTGCTGGTCTGGAGAGATTTCAG |
| MGG-08144DN1R | CGACATCAATCTTGATTCCCAGCACAAC |
| MGG-08144DN2F | GTTGTGCTGGGAATCAAGATTGATGTCG |
| MGG_08144CA/DNOR1 | CGCGGATCCTTAGCAGGCGCATCCATCC |
| GFPR2 | CGACCTGCAGGCATGCAAGCTTTTACTTGTACAGCTCGTCCATGC | Construction of pRP27::GFP vector |
| RP27R | TTTGAAGATTGGGTTCCTACG | Construction of pRP27::MoYpt7-  CA and pRP27::  MoYpt7-DN vectors |
| MGG_08144CA/DNOF2 | CGTAGGAACCCAATCTTCAAAATGTCGTCCAGAAAGAAGGT |
| MGG_08144CA/DNOR2 | CGACCTGCAGGCATGCAAGCTTTTAGCAGGCGCATCCATCC |
| MoVps35OF | CGTAGGAACCCAATCTTCAAAATGGCGTCGGTCCCAGCTCCGC | Construction of pRP27::MoVps35OE vector |
| MoVps35OR | GCTCCTCGCCCTTGCTCACCATCTTGGGATCCAACA |
| Tubulin3F | TCTGACTTCAGGAATGGTCGTTAC | Internal primers for qRT-PCR |
| Tubulin3R | AGCGGTCTGGATGTTGTTGG |
| MoYpt7_CA/DNqRTF | GGAGAGCAAACGGGTGATT | qRT-PCR for MoYpt7-CA、MoYpt7-DN and *MoYPT7* |
| MGG_08144CA/DNqRTR | TACGTTGATGGCCTCCTTTG |
| MGG_05089QF | AACGCAACGAGTCGGTCACA | qRT-PCR for *MoVPS35* |
| MGG_05089QR | GCTTCGGATAAGGTCCAGGG |
| MGG_05089YF | GGCATACGAATTCTTTGCACAGG | Identification of MoVps35-GFP and pRP27::MoVps35-GFP |
| GFPYR | AGTTCACCTTGATGCCGTTCTT |
