# Supplementary material for: The retromer CSC subcomplex is recruited by MoYpt7 and sequentially sorted by MoVps17 for effective conidiation and pathogenicity of the rice blast fungus
Source: Mol Plant Pathol. 2020 Dec 21;22(2):284–98. doi: 10.1111/mpp.13029 (PMC7814966; doi:10.1111/mpp.13029)
Supplement: Supplementary file 5 — TABLE S2 Fungal strains used in this study [file MPP-22-284-s005.doc]

**Table S2 Fungal strains used in this study.**

| Strain | Genotype description | Reference |
| --- | --- | --- |
| Guy11 | Wild type |  |
| *Ku70* | Wild type | (Kershaw MJ et al., 2009) |
| *ΔMoypt7* | MGG_08144 deletion mutant from Guy11 | (Liu XH et al., 2015) |
| *ΔMoypt7-C* | The complemented strain of *ΔMoypt7* | This study |
| *ΔMovps35* | MGG_05089 deletion mutant from *Ku70* | (Zheng et al., 2015) |
| *ΔMovps35-C* | The complemented strain of *ΔMovps35* | (Zheng et al., 2015) |
| *ΔMovps17* | MGG_01434 deletion mutant from *Ku80* | (Zheng et al., 2015) |
| *ΔMovps17-C* | The complemented strain of *ΔMovps17* | (Zheng et al., 2015) |
| *ΔMovps26* | MGG_04830 deletion mutant from *Ku70* | (Zheng et al., 2015) |
| *ΔMovps26-C* | The complemented strain of *ΔMovps26* | (Zheng et al., 2015) |
| *ΔMovps29* | MGG_02524 deletion mutant from *Ku70* | (Zheng et al., 2015) |
| *ΔMovps29-C* | The complemented strain of *ΔMovps29* | (Zheng et al., 2015) |
| *Guy11+MoVps35-GFP* | Guy11 strain expressing MoVps35-GFP construct | This study |
| *MoVps35-flag+RP27-GFP* | Guy11 strain expressing MoVps35-flag and RP27-GFP constructs | This study |
| *MoVps35-flag+ GFP-MoYpt7* | Guy11 strain expressing MoVps35-flag and GFP-MoYpt7 constructs | This study |
| *Guy11+mCherry-MoYpt7+MoVps35-GFP* | Guy11 strain expressing mCherry-MoYpt7 and MoVps35-GFP constructs | This study |
| *Guy11+GFP-MoYpt7+MoVps35- mCherry* | Guy11 strain expressing *GFP-MoYpt7* and *MoVps35- mCherry* constructs | This study |
| *ΔMoypt7+MoVps35-GFP* | *ΔMoypt7* strain expressing MoVps35-GFP construct | This study |
| *Guy11+GFP-MoYpt7* | Guy11 strain expressing GFP-MoYpt7 construct | This study |
| *ΔMovps35+GFP-MoYpt7* | *ΔMovps35* strain expressing GFP-MoYpt7 construct | This study |
| *ΔMovps35+MoYpt7OE* | *ΔMovps35* strain expressing RP27-GFP-MoYpt7 construct | This study |
| *ΔMoypt7+MoVps35OE* | *ΔMoypt7* strain expressing RP27-GFP-MoVps35 construct | This study |
| *GFP-MoYpt7-CA* | Guy11 strain expressing RP27-GFP-MoYpt7CA construct | This study |
| *GFP-MoYpt7-DN* | Guy11 strain expressing RP27-GFP-MoYpt7DN construct | This study |
| *MoVps35-flag+ GFP-MoYpt7CA* | Guy11 strain expressing MoVps35-flag and GFP-MoYpt7CA constructs | This study |
| *MoVps35-flag+ GFP-MoYpt7DN* | Guy11 strain expressing MoVps35-flag and GFP-MoYpt7DN constructs | This study |
| *MoYpt7CA* | Guy11 strain expressing RP27- MoYpt7CA construct | This study |
| *MoYptDN* | Guy11 strain expressing RP27- MoYpt7DN construct | This study |
| *MoYpt7CA+ MoVps35-GFP* | MoYpt7CA strain expressing MoVps35-GFP construct | This study |
| *MoYpt7DN+ MoVps35-GFP* | MoYpt7DN strain expressing MoVps35-GFP construct | This study |
| *Guy11+MoVps17-GFP* | Guy11 strain expressing MoVps17-GFP construct | (Zheng et al., 2017) |
| *ΔMovps35+MoVps17-GFP* | *ΔMovps35* strain expressing MoVps17-GFP construct | (Zheng et al., 2017) |
| *ΔMoypt7+MoVps17-GFP* | *ΔMoypt7* strain expressing MoVps17-GFP construct | (Zheng et al., 2017) |
| *Guy11+MoVps5-GFP* | Guy11 strain expressing MoVps5-GFP construct | (Zheng et al., 2017) |
| *ΔMovps35+MoVps5-GFP* | *ΔMovps35* strain expressing MoVps5-GFP construct | (Zheng et al., 2017) |
| *ΔMoypt7+MoVps5-GFP* | *ΔMoypt7* strain expressing MoVps5-GFP construct | (Zheng et al., 2017) |
| *ΔMovps17+MoVps5-GFP* | *ΔMovps17* strain expressing MoVps5-GFP construct | (Zheng et al., 2017) |
| *ΔMovps17+MoVps35-GFP* | *ΔMovps17* strain expressing MoVps35-GFP construct | (Zheng et al., 2017) |
| *ΔMovps17+GFP-MoYpt7* | *ΔMovps17* strain expressing GFP-MoYpt7 construct | (Zheng et al., 2017) |

Kershaw MJ, Talbot NJ (2009) Genome-wide functional analysis reveals that infection-associated fungal autophagy is necessary for rice blast disease. Proc Natl Acad Sci U S A 106: 15967-15972.

Liu XH, Chen SM, Gao HM, Ning GA, Shi HB, et al. (2015) The small GTPase MoYpt7 is required for membrane fusion in autophagy and pathogenicity of Magnaporthe oryzae. Environ Microbiol 17: 4495-4510.

Zheng W, Zhou J, He Y, Xie Q, Chen A, et al. (2015) Retromer Is Essential for Autophagy-Dependent Plant Infection by the Rice Blast Fungus. PLoS Genet 11: e1005704.

Zheng H, Guo Z, Xi Y, Yuan M, Lin Y, et al. (2017) Sorting nexin (MoVps17) is required for fungal development and plant infection by regulating endosome dynamics in the rice blast fungus. Environ Microbiol 19: 4301-4317.
